# Acute Effects of Cinnamon Spice on Post-prandial Glucose and Insulin in Normal Weight and Overweight/Obese Subjects: A Pilot Study

**DOI:** 10.3389/fnut.2020.619782

**Published:** 2021-01-21

**Authors:** Jing Wang, Sijia Wang, Jieping Yang, Susanne M. Henning, Zahra Ezzat-Zadeh, Shih-Lung Woo, Tianyu Qin, Yajing Pan, Chi-Hong Tseng, David Heber, Zhaoping Li

**Affiliations:** ^1^Center for Human Nutrition, David Geffen School of Medicine, University of California, Los Angeles, Los Angeles, CA, United States; ^2^College of Animal Science and Technology, Hunan Agricultural University, Changsha, China; ^3^Collaborative Innovation Center of Yangtze River Delta Region Green Pharmaceuticals, College of Pharmaceutical Science, Zhejiang University of Technology, Hangzhou, China; ^4^Key Laboratory of Health Cultivation of the Ministry of Education, Beijing University of Chinese Medicine, Beijing, China; ^5^Department of Statistics Core, David Geffen School of Medicine, University of California, Los Angeles, Los Angeles, CA, United States; ^6^Department of Medicine, Veterans Affairs Greater Los Angeles Health Care System, Los Angeles, CA, United States

**Keywords:** cinnamon, insulin, post-prandial, glucose, acute

## Abstract

Clinical studies and meta-analyses have supported the notion that consuming cinnamon spice long term can have beneficial effects in individuals with normal glucose homeostasis and varying degrees of glucose intolerance including type 2 diabetes. The objective of this study was to evaluate the acute effect of cinnamon on the post-prandial responses to a typical American breakfast in normal and overweight/obese participants (ClinicalTrials.gov registration No. NCT04686552). The consumption of a single dose of 6 g of cinnamon added to oatmeal prepared with milk resulted in a significant reduction of one of our primary outcomes post-prandial insulin response (niAUC_0−180min_) in overweight/obese participants compared to control consuming breakfast without cinnamon. We also performed exploratory analysis of secondary outcomes. In normal weight participants, we observed a decrease of post-prandial glucagon response (niAUC_0−180min_ and glucagon levels at 60–120 min) and C-peptide response (30 min) comparing breakfast with to without cinnamon. Cinnamon consumption did not change post-prandial glycemic response in normal weight participants, but increased 60 min post-prandial glucose in overweight/obese participants compared to control. In summary, cinnamon consumption differentially affected post-prandial hormonal responses in normal and overweight/obese participants.

## Introduction

Previous clinical studies and three of four recent meta-analyses have reported beneficial effects of long-term cinnamon intake on blood glucose homeostasis in people with normal glucose homeostasis and varying degrees of glucose intolerance including type 2 diabetes (1–4). For example, in subjects with type 2 diabetes given 1, 3, or 6 g of ground cinnamon per day for 40 days showed significant reductions in fasting serum glucose (18 ~ 29%), triglycerides (23 ~ 30%), LDL cholesterol (7 ~ 27%), and total cholesterol (12 ~ 26%) with no significant changes in the placebo group (5).

Cinnamon (*Cinnamomum verum, Cinnamomum zeylanicum*) and *C. cassia* (*Cinnamomum aromaticum*) have a long history as spices and preservatives (6). Four of the 250 species in the genus Cinnamomum are used as spices and called “cinnamon.” Verum Ceylon or Sri Lankan (also known as *C. zeylanicum*) is often referred to as “true “cinnamon. The three species related to C. cassia, which are more popular, include *C. aromaticum* (also known as Chinese), *C. loureirii* (Saigon or Vietnamese) and *C. burmanni* (Indonesian). In addition, cinnamon is sold in many forms, including harvested sticks of bark (or quills), pulverized bark powder, and extracts derived from the powder. The form in which cinnamon is administered is important because extracts (aqueous and/or organic solvent extraction) and powders made from pulverized bark contain different phytochemicals and may also differ in bioavailability (7).

Various compounds have been identified in different species of cinnamon including cinnamyl alcohol, cinnamaldehyde, cinnamic acid, coumarin, and eugenol (8). *In vitro* and *in vivo* studies suggest that a compound or compounds in the aqueous extract of cinnamon improve insulin sensitivity, glycemic control, and lipid levels by multiple mechanisms including such as activating the insulin receptor by increased auto-phosphorylation, increased glucose transporter-4 (GLUT-4) receptor synthesis and activation, inhibition of pancreatic and intestinal amylase and glucosidase, and increased glycogen synthesis in the liver (9–13). Interest in the potential ability of cinnamon to control glucose in diabetes management increased after the discovery of a bioactive insulin-potentiating agent initially identified as hydroxychalcone derived from cinnamon (7).

Most studies so far have been focused on the long-term health benefit of cinnamon consumption. Improving insulin sensitivity likely contributes to cinnamon mediated glucose lowering effect (7, 14). The knowledge of the acute effect of cinnamon on post-prandial responses is very limited. Postprandial glycemic and hormonal dysregulation are independent risk factors for obesity and cardiovascular diseases (15, 16). The acute effects of cinnamon on the glycemic response were previously evaluated. Supplementation of 50 g available carbohydrate from instant farina cereal with 6 g ground cinnamon lowered post-prandial glycemic response in both normal weight and obese subjects (17). However, the possible mechanism of acute effect of cinnamon on post-prandial glucose, such as its effect on post-prandial insulin secretion using C-peptide as marker, have not been evaluated. The objective of the proposed study is to investigate the acute effect of cinnamon addition to a typical American breakfast on post-prandial glucose, insulin, C-peptide, and glucagon in normal and overweight/obese subjects.

## Materials and Methods

### Study Design

A randomized, controlled, open labeled pilot study with crossover design was carried out at the Center for Human Nutrition, University of California Los Angeles, California, USA. The clinical protocol was approved by the Internal Review Board of the University of California, Los Angeles. All subjects gave written informed consent before enrollment to the study. The study was registered in ClinicalTrials.gov under the following identifier: NCT04686552.

**Figure 1:**
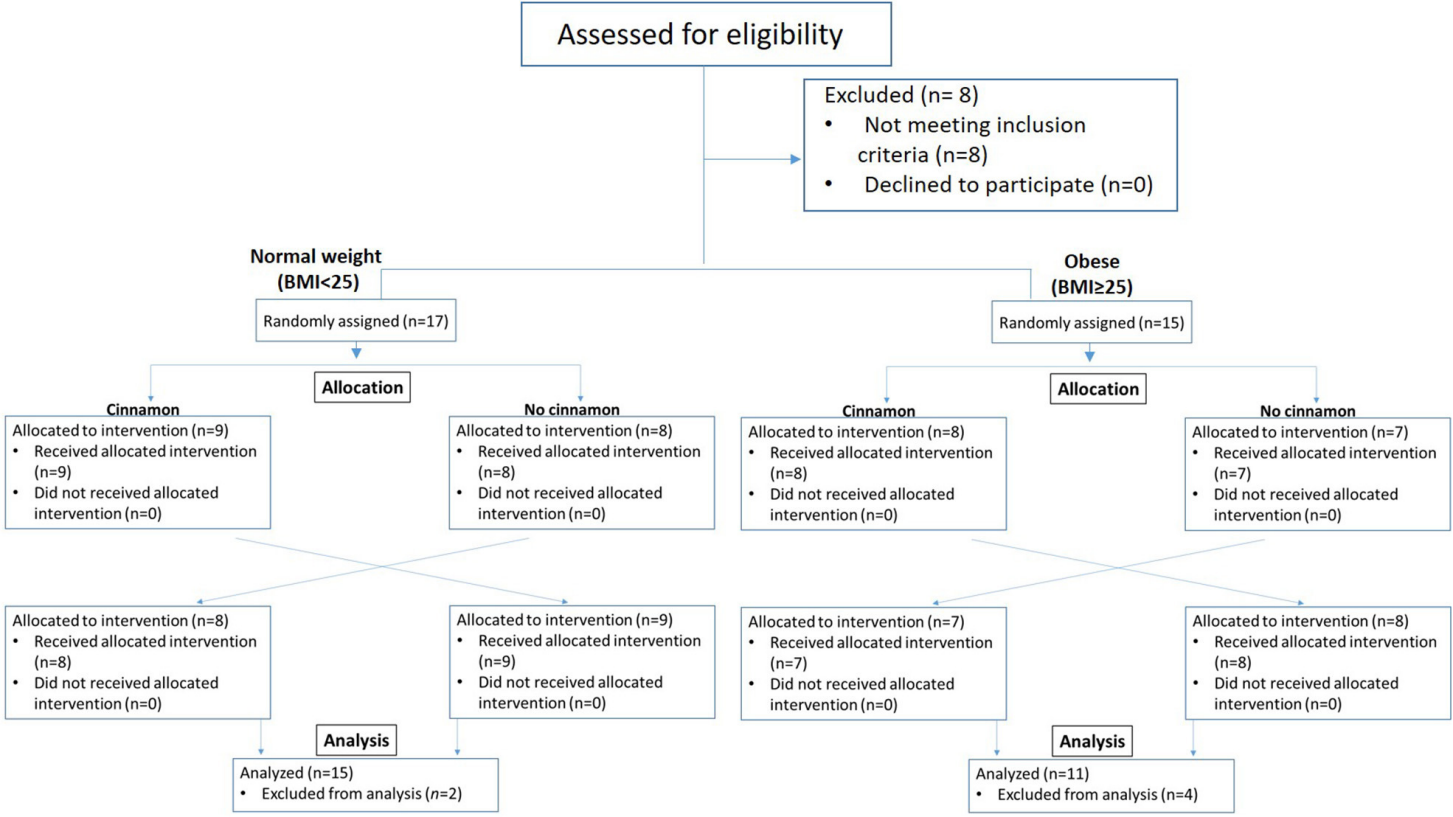
Enrollment, randomization, and analysis of samples of study participants.

Participants were recruited, provided written informed consent and randomized to test meals with or without cinnamon using a computer-generated randomization schedule that was generated by the statistician before the study. The initial screening visit was followed with a 2-week run-in phase. During the 2-week run-in phase, the participants were asked to follow the low fiber/polyphenol diet (beige diet) and exclude cinnamon. Thirty two participants, normal weight (BMI < 25 kg/m^2^, *n* = 17) and overweight/obese subjects (BMI ≥ 25 kg/m^2^, *n* = 15), were randomized to test meals with or without cinnamon in random sequence. On the study day, participants came to the UCLA Center for Human Nutrition in the fasting state and remained for ~4 h. An indwelling catheter was inserted into the vein of the forearm and a baseline (0 h) fasting blood sample collected. The subjects then ate the test meal within 30 min. Blood samples were drawn every 30 min for 3 h after consumption of test meals. There was 1-week washout between two test meals (Figure 1). Participants were asked not to consume any cinnamon or cinnamon products during washout. Weight and body composition were measured after participants arrived at the Center for Human Nutrition at each visit after resting for 15 min. Body weight and body composition was determined using the Tanita-BC418 body-fat analyzer (Tanita Corp., Tokyo, Japan). The instrument uses bioelectric impedance analysis to determine body composition and a digital scale for body weight.

### Participants

Study participants met the enrollment criteria (1) age 20–50 year old; (2) typically consume low fiber/polyphenol diet (beige diet). Participants who were taking blood thinning medications such as Warfarin or Coumadin, eating a high fiber/polyphenol diet (≥10 g fibers and ≥3 servings of polyphenol rich fruit/vegetables) were excluded. Seventeen normal weight (BMI 18.5–24.9 kg/m^2^) and fifteen overweight/obese participants (BMI > 25 kg/m^2^) were enrolled and completed the intervention.

### Test Meals

The test meal consisted of ½ cup dry instant oatmeal, prepared with 1 cup of 2% milk served with or without 6 g of ground cinnamon. The total carbohydrate, accounting the oatmeal and milk, is 50 g. The oatmeal used was a commercially available maple and brown sugar Quaker Instant Oatmeal. This ready-made instant oatmeal contained 12 g of sugar per meal. The added sugar was included in the calculation of the carbohydrate content. This is a commonly consumed breakfast in the United States. Although the instant oatmeal contains 3 g of dietary fiber (1 g soluble, 2 g insoluble) including beta-glucan, the quickly digestible form of oatmeal combined with added sugar presents a high glycemic meal. Six gram cinnamon was previously reported to acutely lower post-prandial glycemic response (17). In this study we used Korintje cinnamon, ground from cassia bark. This cinnamon is the most widely available cinnamon in the United States. Cinnamon and oatmeal were purchased from a local grocery store.

### Blood Biochemical Analyses

Serum glucose was determined using the Cayman Glucose Assay (Cayman Chemical Company, Ann Arbor, MI) kit based on a colorimetric determination using the glucose oxidase-peroxide reaction. Serum insulin, C-peptide, glucagon were determined using a Luminex kit (EMD Millipore, Billerica, MA) with the MAGPIX multiplexing system. Detection and quantitation were based on fluorescent readings from the MagPix and Milliplex software.

### High-Performance Liquid Chromatography (HPLC) Cinnamon Analysis

The HPLC/photodiode array detector analysis was performed on Zorbax C18 4.6 × 50 mm Agilent column. The mobile phase for HPLC analysis consisted of two solutions: (A) Gradient from 5% Acetonitrile to 50% Acetonitrile and (B) 95 to 50% 0.2 % Formic acid/H_2_O in 30 min. Peak identification was based on retention time at 280 nm Concentrations were calculated by comparison of sample peak area with the commercial standard peak area. Cinnamic acid and cinnamaldehyde standards were purchased from Sigma-Aldrich (St. Louis, MO).

### Statistical Analyses

A previous study has demonstrated that 6 g cinnamon intake effectively reduced post-prandial glycemic response in normal and obese participants with an effect size of 0.73 (17). Using this data we estimated that a sample of 24–25 subjects (with allowance of 10% dropout rate) will provide 80% power to detect an effect size of 0.73 for the primary endpoints with type I error of 2.5%. A sample of 17 subjects will provide 81% power and a sample of 15 subjects will provide 75% power with type I error of 5 % to detect the same effect size differences in glycemic response to between cinnamon and control groups. This calculation for subgroups was not adjusted for multiple comparisons.

The primary outcomes are post-prandial glycemic and insulin responses. Secondary outcomes are post-prandial C-peptide and glucagon responses. Total area under the curve (AUC) is known to strongly correlate with basal blood glucose value and incremental AUC (iAUC) more accurately describe glycemic response to foods (18). Due to the wide variations in baseline blood glucose, insulin, C-peptide and glucagon, The net incremental area under curve (niAUC_0−180min_) over the 3 h was calculated using the niAUC (apply trapezoid rule for all increments positive and negative) as previously described (19).

Mann-Whitney's tests and Fisher's exact tests were used to analyze differences in the baseline characteristics between normal weight and overweight/obese participants. niAUC_0−180min_ as well as changes of glucose, insulin, C-peptide, and glucagon at each time point from baseline (time 0) were compared between cinnamon and control using linear mixed effects models. Covariates age, sex, race, and BMI were included as fixed effects, and subjects as a random effect. The primary endpoint analyses of glycemic and insulin response used Bonferroni correction to account for multiple comparisons, and a *p* < 0.025 was considered statistically significant. Analyses of secondary endpoints and subgroup analyses were considered exploratory and no multiple comparison adjustment was made. All Statistical analysis were performed using R (www.r-project.org).

## Results

### Analysis of Cinnamon

**Figure 2:**
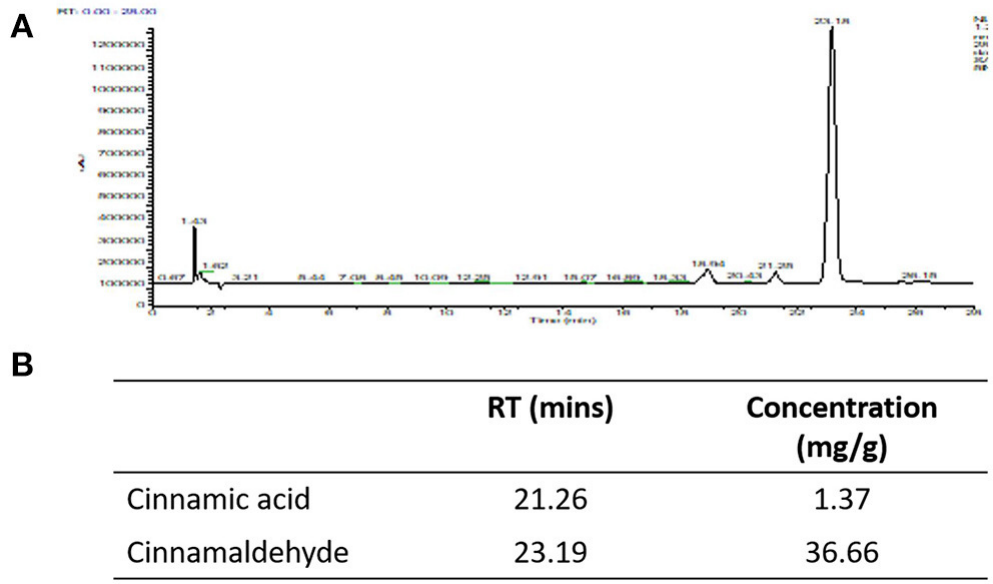
HPLC was utilized to quantify cinnamic acid and cinnamaldehyde. **(A)** HPLC chromatograms, **(B)** concentrations of cinnamic acid and cinnamaldehyde in the cinnamon powder used in this study.

Our HPLC analysis showed that each gram of Korintje cinnamon used in this study contains 1.37 mg cinnamic acid and 36.66 mg cinnamaldehyde (Figure 2).

### Characteristics of Study Participants

Seventeen normal weight participants, age 20 to 43 years, 15 overweight participants, age 23 to 50 years, completed the study. In this study, 6 participants were excluded from the data analysis due to large intra-individual variation (>10%) of fasting blood glucose among 2 visits. Two of the six participants were in the normal weight group and 4 in the overweight/obese group. High intra-individual fasting blood glucose variability are associated with impaired glucose homeostasis and T2DM (20, 21) and concern of compliance with fasting instruction. We therefore performed data analysis in participants with more stable and less variance in fasting blood glucose (CV <10%, Table 1). Normal weight and overweight/obese participants were similar in sex and fasting insulin, differed significantly in age (*P* = 0.008), weight (*P* = 0.012), BMI (*P* = 0.000), fasting glucose (*P* = 0.000), C-peptide (*P* = 0.000), and glucagon (*P* = 0.027).

**Table 1:** Demographics and laboratory characteristics of study participants with stable fasting blood glucose^*^.

	**All (*n* = 26)**	**Normal weight (*n* = 15)**	**Overweight/Obese (*n* = 11)**	***P-*value (Normal weight vs. Overweight/Obese)**
Sex, % women	50.0%	46.7%	54.5%	NS
Age, y	30.6 ± 9.9	26.3 ± 6.8	37.1 ± 10.6	0.008
Weight, kg	71.4 ± 15.9	63.5 ± 9.2	82.0 ± 17.2	0.012
BMI, kg/m^2^	25.2 ± 4.5	22.0 ± 1.6	29.6 ± 3.4	0.000
Fasting glucose, mg/dL	90.4 ± 16.1	81.9 ± 5.5	102.2 ± 18.2	0.000
Fasting insulin, pg/mL	1,045.4 ± 1,083.2	1,427.2 ± 1,307.1	524.9 ± 145.1	NS
Fasting C-peptide, pg/mL	1,042.6 ± 492.1	735.7 ± 190.1	1,468.1 ± 464.0	0.000
Fasting Glucagon, pg/mL	40.5 ± 33.2	49.4 ± 35.6	28.3 ± 24.4	0.027

* *Data are means ± SDs; Fisher's exact test and Mann–Whitney's test were used to compare the sex, age, weights, BMI between normal weight and overweight/obese participants*.

### Post-prandial Serum Glucose, Insulin, C-peptide, and Glucagon Responses to Test Meals in Normal Weight and Overweight/Obese Participants

**Figure 3:**
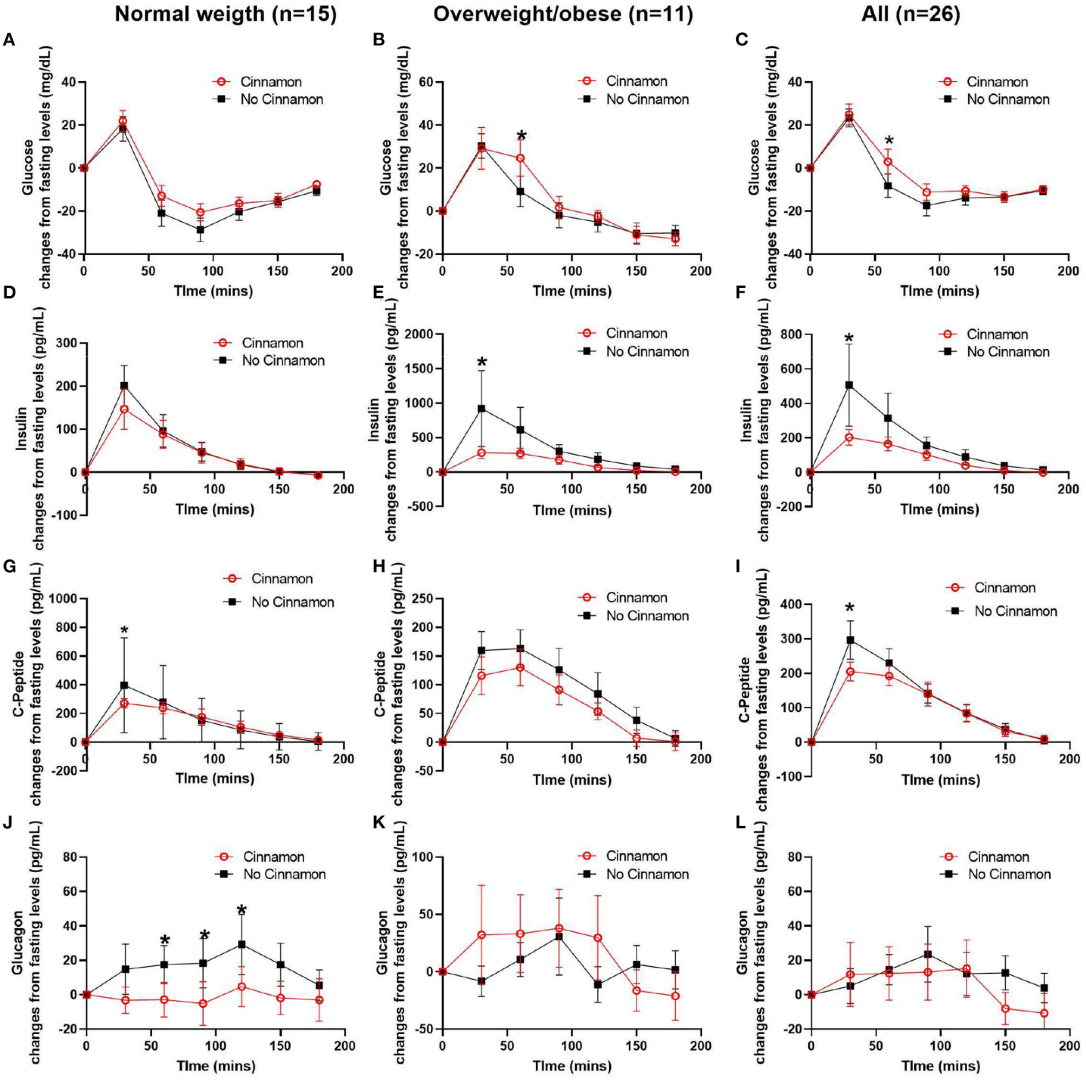
Changes of serum glucose, C-peptide, insulin, and glucagon from fasting levels in normal weight (*n* = 15), overweight/obese (*n* = 11) participants, and all participants (*n* = 26). **(A–C)** glucose, **(D–F)** insulin, **(G–I)** C-peptide, and **(J–L)** glucagon, Data are mean ± SEMs, *Results were significant when *P* < 0.05 for comparison between cinnamon and group at each time point.

We performed post-prandial glycemic and insulin responses analyses as the primary outcomes, as well as exploratory analyses of C-peptide and glucagon responses as our secondary outcomes in 15 normal weight and 11 overweight/obese participants with stable fasting blood glucose after consuming the test meal with or without 6 g cinnamon (Table 2, Figure 3).

**Table 2:** Glucose, insulin, C-peptide, and glucagon niAUC_0−180min_ [mean (SD)] in participants with stable fasting blood glucose^*^.

	**Normal weight (*****n*** **=** **15)**			**Overweight/Obese (*****n*** **=** **11)**			**All (*****n*** **=** **26)**		
	**Cinnamon**	**Control**	***P***	**Cinnamon**	**Control**	***P***	**Cinnamon**	**Control**	***P***
Glucose niAUC_0−180min_ (mg.min/dL)	−1,140 (1,186)	−1,800 (1,533)	NS	740 (2,609)	253 (2,099)	NS	−344 (2,100)	−932 (2,036)	NS
Insulin niAUC_0−180min_ (pg.min/mL)	54,471 (77,739)	66,183 (45,725)	NS	125,112 (93,356)	153,852 (103,040)	0.001	84,357 (90,206)	103,274 (85,841)	0.024
C-peptide niAUC_0−180min_ (pg.min/mL)	144,836 (91,037)	156,040 (83,540)	NS	142,364 (127,252)	178,420 (925,14)	NS	143,790 (105,451)	165,509 (86,365)	NS
Glucagon niAUC_0−180min_ (pg.min/mL)	−853 (1,905)	347 (1,905)	0.001	−370 (1,832)	−272 (1,643)	NS	−648 (1,853)	85 (1,791)	0.023

* *NS, no significance; niAUC_0−180min_ were compared between cinnamon and control using linear mixed effects models. Covariates age, sex, race, and BMI were included as fixed effects, and subjects as a random effect*.

In normal weight participants (*n* = 15), the post-prandial glucagon niAUC_0−180min_ and glucagon levels at 60, 90, and 120 min post-prandially were lower after meal with cinnamon compared to test meal without cinnamon (Table 2, Figure 3J). In addition, serum C-peptide at 30 min was decreased with cinnamon compared to without (Figure 3G). No significant difference was observed in niAUC_0−180_ for glucose, insulin and C-peptide between test meals with and without cinnamon (Table 2). AUC_0−180min_ were similar between cinnamon and control (no cinnamon) groups for all the markers evaluated in normal weight participants (Supplementary Table 1).

In overweight/obese participants (*n* = 11), post-prandial insulin niAUC_0−180min_ and serum insulin at 30 min were significantly lower with cinnamon compared to test meal without cinnamon (Table 2, Figure 3E). In addition, in overweight/obese participants, we did not detect significant differences between two test meals in niAUC_0−180min_ for glucose, C-peptide and glucagon (Table 2). Blood glucose at 60 min, however, was higher in overweight/obese participants consuming oatmeal with cinnamon compared to without (Figure 3B). AUC_0−180min_ were similar between cinnamon and control for all the markers evaluated in overweight/obese participants (Supplementary Table 1).

When all subjects were combined (*n* = 26), post-prandial insulin and glucagon niAUC_0−180min_ remained lower after meal with cinnamon compared to test meal without cinnamon (Table 2). Insulin and C-peptide levels at 30 min were lower while glucose level at 60 min were higher after meal with cinnamon compared to test meal without cinnamon (Figures 3C,F,I).

## Discussion

The health benefit of long-term cinnamon consumption has been previously evaluated (1–5, 22). In Type 2 diabetes, the glucose lowering effects of long-term cinnamon supplementation has been consistently reported (22, 23). However, current knowledge of cinnamon's acute effect is very limited (17, 24). Post-prandial glucose and insulin dysregulation are independent risk factor for obesity and cardiovascular diseases (15, 16). Therefore, in this pilot study we aimed to evaluate the acute effect of cinnamon on post-prandial glycemic and insulin responses as our primary outcomes. The results of present study need to be carefully interpreted due to the small sample size. We observed that the addition of 6 g cinnamon to an oatmeal/milk breakfast decreased serum insulin (niAUC_0−180min_ and 30 min) in overweight/obese participants compared to a control meal without cinnamon. In addition, we performed exploratory evaluation of post-prandial C-peptide and glucagon response as our secondary outcomes. We observed that the addition of 6 g cinnamon to an oatmeal/milk breakfast decreased serum glucagon (niAUC_0−180min_ and 60–120 min) in normal weight participants compared to a control meal without cinnamon. We did not observe significant decrease in serum glucose as previously observed by Magistrelli and Chezem (17) and Solomon and Blannin (24), while serum glucose was increased post-prandially at 60 min in overweight/obese participants.

In the study by Magistrelli and Chezem (17) the acute effect of cinnamon was evaluated on post-prandial glucose response in both normal weight and obese subjects in a similar study design. They found that 6 g of cinnamon reduced post-prandial glucose at 1 h but it was increased at 2 h. The other study of acute cinnamon consumption by Solomon et al. also found that intake of 5 g of cinnamon acutely reduced blood glucose levels during OGTT test as well as improved insulin sensitivity (24). However, in our study the blood glucose was not changed by cinnamon in normal weight participants and was elevated at 60 min during meal challenge in overweight/obese participants. One major difference between our study and others are the test meal composition. The other two groups evaluated the acute effect of cinnamon with either instant cereal or glucose as the only carbohydrate sources, while the carbohydrates of our test meal include not only oatmeal but also 1 cup of 2% fat milk. Bovine milk contains about 4.8% lactose and whether cinnamon affects milk lactose intestinal breakdown to glucose is unknown and could potentially affect the blood glucose profiles. Additional differences in the test meals include the fiber content of oatmeal as well as the added sucrose in the oatmeal. A previous study showed that fiber content and type have an impact on glucose and insulin response (25). Sucrose is ~50% glucose and 50% fructose and have a smaller impact on glycemia than pure glucose. A previous study demonstrated that berry intake has an effect on post-prandial glucose response compared to sucrose intake (26). Further investigations of the effect of cinnamon on intestinal breakdown of lactose to glucose as well as the contribution of fiber and sucrose content will help to understand if these are the potential underlying mechanisms contributing to the lack of effect on serum glucose and slightly higher 60 min post-prandial glucose concentration observed in overweight/obese participants.

Another difference between our pilot study and the other two studies are the characteristics of study participants. Although Magistrelli and Chezen (17) evaluated the acute effect of cinnamon in both normal weight and obese subjects, the obese subjects in their study were young, healthy and had similar fasting glucose as normal weight subjects. The cinnamon-induced post-prandial glucose-lowering response was observed only when they combined the data from both normal weight and obese subjects. Solomon et al. evaluated the acute effect of cinnamon in glucose control only in young healthy subjects. Consistent with their observation, we found that cinnamon induced a decrease in 30 min post-prandial C-peptide (*P* = 0.03) and insulin (*P* = 0.06) without affecting glucose response in normal weight participants, suggesting cinnamon may acutely regulate glucose stimulated-insulin secretion or improve insulin sensitivity.

In this pilot study, overweight/obese participants were older, and fasting blood glucose was significantly higher than in normal weight participants (81.9 ± 5.5 mg/dL vs. 102.2 ± 18.2 mg/dL, *P* < 0.01), suggesting impaired glucose homeostasis in these participants. In pre-diabetes, individuals present with only slightly elevated blood glucose levels as the pancreas seeks to compensate with increased insulin secretion for many years until type 2 diabetes mellitus is diagnosed (27, 28). The acute effect of cinnamon on reducing 30 min post-prandial insulin and insulin niAUC may spares the pancreatic beta-cell and therefore might slow the progression of pre-diabetes to type 2 diabetes (5).

Glucagon, a peptide hormone induces the opposite regulatory effect compared to insulin. Glucagon stimulates hepatic glucose formation during fasting (29). Previous studies showed that oatmeal intake induced positive post-prandial glucagon responses in both overweight and T2DM subjects (25, 30, 31). In the present study no change in post-prandial glucagon was observed in response to the test meals in both normal weight and overweight/obese subjects. However, cinnamon supplementation improved post-prandial glucagon suppression in normal weight subjects. A failure to suppress post-prandial glucagon concentrations is often observed in T2DM (32). Therefore, normal weight individuals might be more responsive toa decrease in post-prandial glucagon compared to overweight/obese individuals (29). Whether the effect of cinnamon on post-prandial glucagon mechanistically is also related to improved glucose metabolism during long-term cinnamon consumption, needs further investigation.

The major limitation of this pilot study is small sample size. Future studies should have larger subject numbers with uniform characteristics, such as age, for normal weight, and overweight/obese participants. The second limitation is the open label design, which may lead to bias. Although results from the current study did not support the acute hypoglycemic effect of cinnamon in both normal weight and overweight/obese participants, the significant decrease of post-prandial insulin response without increased post-prandial glycemic response in overweight/obese participants support the previously observed potential of cinnamon to improve insulin sensitivity (7, 14). However, we observed a temporarily higher post-prandial 1 h glucose, which might be critical for this high-risk population. It is therefore important to perform additional clinical trials with combined acute and chronic design to understand if cinnamon's acute post-prandial effect can predict its health outcomes after long-term consumption.

## Data Availability Statement

The original contributions presented in the study are included in the article/Supplementary Material, further inquiries can be directed to the corresponding author/s.

## Ethics Statement

The studies involving human participants were reviewed and approved by Human Subjects Protection Committee of the University of California, Los Angeles Internal Review Board of the University of California, Los Angeles. The patients/participants provided their written informed consent to participate in this study.

## Author Contributions

ZL, DH, and SMH designed the study. ZE-Z, SW, JW, YP, and TQ conducted the research. JY, JW, and S-LW analyzed the data. C-HT performed the statistical analysis. JY and JW wrote the manuscript. All authors read, edited, and approved the final manuscript.

## Conflict of Interest

The authors declare that the research was conducted in the absence of any commercial or financial relationships that could be construed as a potential conflict of interest.

## Abbreviations

GLUT-4: glucose transporter-4
AUC: area under curve
niAUC: net incremental area under curve
LDL: low-density lipoprotein
BMI: body mass index
HPLC: high performance liquid chromatograph
CV: coefficient of variation
OGTT: oral glucose tolerance test
T2DM: type-II diabetes mellitus.

## References

[1] Davis PA Yokoyama W. Cinnamon intake lowers fasting blood glucose: meta-analysis. J Med Food. (2011) 14:884–9. 10.1089/jmf.2010.0180 21480806

[2] Leach MJ Kumar S. Cinnamon for diabetes mellitus. Cochrane Database Syst Rev. (2012) 2012:CD007170. 10.1002/14651858.CD007170.pub2 22972104 PMC6486047

[3] Allen RW Schwartzman E Baker WL Coleman CI Phung OJ. Cinnamon use in type 2 diabetes: an updated systematic review and meta-analysis. Ann Family Med. (2013) 11:452–9. 10.1370/afm.1517 24019277 PMC3767714

[4] Anderson RA Zhan Z Luo R Guo X Guo Q Zhou J. Cinnamon extract lowers glucose, insulin and cholesterol in people with elevated serum glucose. J Tradit Complement Med. (2016) 6:332–6. 10.1016/j.jtcme.2015.03.005 27774415 PMC5067830

[5] Khan A Safdar M Khan MMA Khattak KN Anderson RA. Cinnamon improves glucose and lipids of people with type 2 diabetes. Diabetes Care. (2003) 26:3215–8. 10.2337/diacare.26.12.3215 14633804

[6] Rao PV Gan SH. Cinnamon: a multifaceted medicinal plant. Evid Based Complement Alternat Med. (2014) 2014:642942. 10.1155/2014/642942 24817901 PMC4003790

[7] Cheng DM Kuhn P Poulev A Rojo LE Lila MA Raskin I. *In vivo* and *in vitro* antidiabetic effects of aqueous cinnamon extract and cinnamon polyphenol-enhanced food matrix. Food Chem. (2012) 135:2994–3002. 10.1016/j.foodchem.2012.06.117 22980902 PMC3444749

[8] Avula B Smillie TJ Wang YH Zweigenbaum J Khan IA. Authentication of true cinnamon (Cinnamon verum) utilising direct analysis in real time (DART)-QToF-MS. Food Addit Contam Part A Chem Anal Control Expo Risk Assess. (2015) 32:1–8. 10.1080/19440049.2014.981763 25421162

[9] Cao H Polansky MM Anderson RA. Cinnamon extract and polyphenols affect the expression of tristetraprolin, insulin receptor, and glucose transporter 4 in mouse 3T3-L1 adipocytes. Arch Biochem Biophys. (2007) 459:214–22. 10.1016/j.abb.2006.12.034 17316549

[10] Qin B Dawson HD Schoene NW Polansky MM Anderson RA. Cinnamon polyphenols regulate multiple metabolic pathways involved in insulin signaling and intestinal lipoprotein metabolism of small intestinal enterocytes. Nutrition. (2012) 28:1172–9. 10.1016/j.nut.2012.03.020 22858201

[11] Ranasinghe P Jayawardana R Galappaththy P Constantine G De Vas Gunawardana N Katulanda P. Efficacy and safety of ‘true’cinnamon (*Cinnamomum zeylanicum*) as a pharmaceutical agent in diabetes: a systematic review and meta-analysis. Diabet Med. (2012) 29:1480–92. 10.1111/j.1464-5491.2012.03718.x 22671971

[12] Beejmohun V Peytavy-Izard M Mignon C Muscente-Paque D Deplanque X Ripoll C. Acute effect of *Ceylon cinnamon* extract on postprandial glycemia: alpha-amylase inhibition, starch tolerance test in rats, and randomized crossover clinical trial in healthy volunteers. BMC Complement Alternat Med. (2014) 14:351. 10.1186/1472-6882-14-351 25249234 PMC4246455

[13] Medagama AB. The glycaemic outcomes of *Cinnamon*, a review of the experimental evidence and clinical trials. Nutr J. (2015) 14:108. 10.1186/s12937-015-0098-9 26475130 PMC4609100

[14] Sartorius T Peter A Schulz N Drescher A Bergheim I Machann J. Cinnamon extract improves insulin sensitivity in the brain and lowers liver fat in mouse models of obesity. PLoS ONE. (2014) 9:e92358. 10.1371/journal.pone.0092358 24643026 PMC3958529

[15] Blaak E Antoine JM Benton D Björck I Bozzetto L Brouns F. Impact of postprandial glycaemia on health and prevention of disease. Obes Rev. (2012) 13:923–84. 10.1111/j.1467-789X.2012.01011.x 22780564 PMC3494382

[16] Berry SE Valdes AM Drew DA Asnicar F Mazidi M Wolf J. Human postprandial responses to food and potential for precision nutrition. Nat Med. (2020) 26:964–73. 10.1038/s41591-020-0934-0 32528151 PMC8265154

[17] Magistrelli A Chezem JC. Effect of ground cinnamon on postprandial blood glucose concentration in normal-weight and obese adults. J Acad Nutr Diet. (2012) 112:1806–9. 10.1016/j.jand.2012.07.037 23102179

[18] Le Floch J-P Escuyer P Baudin E Baudon D Perlemuter L. Blood glucose area under the curve: methodological aspects. Diabet Care. (1990) 13:172–5. 10.2337/diacare.13.2.172 2351014

[19] Brouns F Bjorck I Frayn K Gibbs A Lang V Slama G. Glycaemic index methodology. Nutrition research reviews. (2005) 18:145–71. 10.1079/NRR2005100 19079901

[20] Ollerton RL Playle R Ahmed K Dunstan FD Luzio SD Owens DR. Day-to-day variability of fasting plasma glucose in newly diagnosed type 2 diabetic subjects. Diabet Care. (1999) 22:394–8. 10.2337/diacare.22.3.394 10097916

[21] Krüger L Slabber M Joubert G Venter CS Vorster HH Intra-and inter-individual variation in blood glucose response to white bread and glucose in patients with type 2 diabetes mellitus. S Afr J Clin Nutr. (2003) 16. Available online at: http://www.sajcn.co.za/index.php/SAJCN/article/view/30

[22] Santos HO Da Silva GA. To what extent does cinnamon administration improve the glycemic and lipid profiles? Clin Nutr ESPEN. (2018) 27:1–9. 10.1016/j.clnesp.2018.07.011 30144878

[23] Costello RB Dwyer JT Saldanha L Bailey RL Merkel J Wambogo E. Do cinnamon supplements have a role in glycemic control in type 2 diabetes? A narrative review. J Accad Nutr Diet. (2016) 116:1794–802. 10.1016/j.jand.2016.07.015 27618575 PMC5085873

[24] Solomon T Blannin A. Effects of short-term cinnamon ingestion on *in vivo* glucose tolerance. Diabetes Obes Metab. (2007) 9:895–901. 10.1111/j.1463-1326.2006.00694.x 17924872

[25] Behall KM Scholfield DJ Hallfrisch J. Comparison of hormone and glucose responses of overweight women to barley and oats. J Am Coll Nutr. (2005) 24:182–8. 10.1080/07315724.2005.10719464 15930484

[26] Torronen R Sarkkinen E Tapola N Hautaniemi E Kilpi K Niskanen L. Berries modify the postprandial plasma glucose response to sucrose in healthy subjects. Br J Nutr. (2010) 103:1094–7. 10.1017/S0007114509992868 19930765

[27] Mcgarry JD. Banting lecture 2001: dysregulation of fatty acid metabolism in the etiology of type 2 diabetes. Diabetes. (2002) 51:7–18. 10.2337/diabetes.51.1.7 11756317

[28] Tabák AG Jokela M Akbaraly TN Brunner EJ Kivimäki M Witte DR. Trajectories of glycaemia, insulin sensitivity, and insulin secretion before diagnosis of type 2 diabetes: an analysis from the Whitehall II study. Lancet. (2009) 373:2215–21. 10.1016/S0140-6736(09)60619-X 19515410 PMC2726723

[29] Geary N. Postprandial suppression of glucagon secretion: a puzzlement. Diabetes. (2017) 66:1123–5. 10.2337/dbi16-0075 28507213

[30] Mottalib A Mohd-Yusof BN Shehabeldin M Pober DM Mitri J Hamdy O. Impact of diabetes-specific nutritional formulas versus oatmeal on postprandial glucose, insulin, GLP-1 and postprandial lipidemia. Nutrients. (2016) 8:443. 10.3390/nu8070443 27455318 PMC4963919

[31] Mottalib A Abrahamson MJ Pober DM Polak R Eldib AH Tomah S. Effect of diabetes-specific nutrition formulas on satiety and hunger hormones in patients with type 2 diabetes. Nutr Diabet. (2019) 9:1–6. 10.1038/s41387-019-0093-x 31551412 PMC6760115

[32] Meier JJ Kjems LL Veldhuis JD Lefèbvre P Butler PC. Postprandial suppression of glucagon secretion depends on intact pulsatile insulin secretion: further evidence for the intraislet insulin hypothesis. Diabetes. (2006) 55:1051–6. 10.2337/diabetes.55.04.06.db05-1449 16567528

