# Supplementary material for: Acute Effects of Cinnamon Spice on Post-prandial Glucose and Insulin in Normal Weight and Overweight/Obese Subjects: A Pilot Study
Source: Front Nutr. 2021 Jan 21;7:619782. doi: 10.3389/fnut.2020.619782 (PMC7859251; doi:10.3389/fnut.2020.619782)
Supplement: Supplementary file 1 [file Table_1.DOCX]

**Supplementary Table 1:** Glucose, insulin, C-peptide, glucagon and triglycerides AUC over 3h (mean (SD)) in participants with stable fasting blood glucose

|  | Normal Weight (n=15) | | | Overweight/Obese (n=11) | | |
| --- | --- | --- | --- | --- | --- | --- |
|  | Cinnamon | Control | *P* | Cinnamon | Control | *P* |
| Glucose AUC_0-180min_ | 13264(1588) | 12595(1438) | NS | 19458(2848) | 18555(3089) | NS |
| Insulin  AUC_0-180min_ | 313533(216198) | 320900(230187) | NS | 222340(100544) | 245586(106715) | NS |
| C-peptide AUC_0-180min_ | 278036(101199) | 287690(89665) | NS | 409245(116348) | 440043(110073) | NS |
| Glucagon AUC_0-180min_ | 8214(5524) | 9049(6792) | NS | 4402(3947) | 5132(4078) | NS |
